# Investigating Coenzyme Function of Thiamine Triphosphate Using Its Novel Hydrolysis-Resistant Analog and Transketolase

**DOI:** 10.3390/biom16020304

**Published:** 2026-02-14

**Authors:** Artem V. Artiukhov, Alexey V. Kazantsev, Olga N. Solovjeva, Vasily A. Aleshin

**Affiliations:** 1 Belozersky Institute of Physico-Chemical Biology, Lomonosov Moscow State University, 119991 Moscow, Russia; 2 Department of Biological Chemistry, Sechenov University, 119048 Moscow, Russia; 3 Faculty of Chemistry, Lomonosov Moscow State University, 119234 Moscow, Russia

**Keywords:** thiamine triphosphate, bismethylene thiamine triphosphate, thiamine diphosphate, transketolase, thiamine coenzyme function, hydrolysis-resistant analog, vitamin synthetic analog, coenzyme docking

## Abstract

Thiamine (vitamin B1) and its phosphates are essential for almost all organisms. Thiamine diphosphate (ThDP) is the major intracellular derivative which is considered the only form functioning as a coenzyme. Thiamine triphosphate (ThTP), another ubiquitous derivative, lacks a clear physiological function and is usually kept at low levels. However, it can accumulate up to 87% of total thiamine in animal tissues lacking cytosolic thiamine triphosphatase (THTPA) activity. Studies of ThTP coenzyme function have always faced the problem of ThTP hydrolysis to ThDP. To avoid such interference a synthetic stable ThTP analog, bismethylene ThTP (bmThTP), has been synthesized. Given that ThTP accumulation is caused by cytosolic THTPA suppression, cytosolic ThDP-dependent transketolase (TKT) is the primary target for probing (bm)ThTP’s coenzyme function. Indeed, bmThTP acts as a TKT coenzyme, with the apparent K_m_(bmThTP) of 16.3 µM. However, bmThTP binding slightly differs from that of ThTP. Molecular docking was used to estimate affinities of ThDP, ThTP and bmThTP, also allowing us to avoid ThTP hydrolysis. Despite almost identical localization within the active site, bmThTP could not bind as well as ThTP, resulting in a 2.36 kcal/mol difference in estimated ΔG. Based on our data, calculated K_m_(ThTP) for TKT is about 0.07–0.08 µM, only 1.6–2 times that of K_m_(ThDP). Such a small difference implies that ThTP could physiologically act as the main TKT coenzyme form upon its accumulation in muscles, at least in a few known animal species.

## 1. Introduction

Natural derivatives of thiamine (vitamin B1) include a number of its phosphates and phosphoadenylated forms–thiamine mono-(ThMP), di-(ThDP) and triphosphate (ThTP) as well as adenosine ThTP and adenosine ThDP (AThTP and AThDP, respectively) [1]. These forms and thiamine itself are found in almost all organisms, with only extremely rare exceptions such as parasitic bacterium *Borrelia burgdorferi* [2]. ThDP is undoubtedly the main form of all thiamine derivatives as it enables the essential coenzyme function of thiamine. That is, ThDP is required for a large group of enzymes to catalyze their reactions [1,3]. ThDP is considered the only coenzyme derivative of thiamine, although other forms have been probed for this ability [4]. For some time there was even uncertainty whether ThTP could act as a coenzyme of pyruvate decarboxylase (EC: 4.1.1.1); however, contamination of thiamine triphosphatase was shown to be responsible for the production of ThDP from ThTP [5,6,7,8].

ThTP is a ubiquitous thiamine derivative which lacks a clear physiological role [9,10]. Its concentrations are normally kept at very low levels, accounting for just about 1% of total thiamine in rat tissues [11]. The latter results from the activity of cytosolic thiamine triphosphatase (THTPA); however, ThTP may accumulate without negative consequences in tissues devoid of THTPA activity such as pig skeletal muscle [12], chicken skeletal muscle [13] or the electric organ of Electrophorus electricus [11]. ThTP content may gain up to 87% of the total thiamine pool, thus being the principal thiamine derivative in the cell [11]. In view of the cases of such a large ThTP accumulation in animals, here we aimed to revisit the potential role of ThTP as the coenzyme.

There are five enzymes relying on ThDP as the coenzyme in animals, or eleven ThDP-dependent genes including all paralogs. They are transketolase (TKT), pyruvate dehydrogenase (PDH), 2-oxoglutarate dehydrogenase (OGDH), branched-chain α-keto acid dehydrogenase (BCDH), and 2-hydroxyacyl-CoA lyase (HACL) [3]. While PDH, OGDH, and BCDH are primarily localized in mitochondria [14,15], and two HACL isoenzymes are localized to peroxisomes and the endoplasmic reticulum [16,17], TKT is localized to cytosol [18]. Considering the cytosolic localization of THTPA, whose activity suppression can be responsible for the accumulation of ThTP [19], TKT appears as the primary target to probe for the ThTP coenzyme function.

To avoid interference from ThTP hydrolysis [5,6,7,8], several ThTP analogs in which phosphate groups are replaced with similar ones, can be synthesized. Such analogs have not been described for ThTP itself, but many are shown for ATP, among which are methylene, imino, thiophosphate, vanadate as well as aluminum, beryllium and magnesium fluoride derivatives [20]. However, the methylene analog of ATP seems the most resistant to enzymatic hydrolysis, adopting a narrower range of conformations and better corresponding to ATP rather than transition state analogs of ATP hydrolysis [20]. Thus we have decided to synthesize a bismethylene ThTP analog (bmThTP) and analyze its effect on TKT.

## 2. Materials and Methods

### 2.1. Chemicals

Chemicals were obtained from Sigma-Aldrich (Steinheim, Germany) unless otherwise specified. Thiamine hydrochloride was purchased from Sisco Research Laboratories Pvt. Ltd. (Mumbai, India), ThDP was from Solarbio (Beijing, China), NADH was from Macklin Biochemical (Shanghai, China), silica gel C18 was from Hawach Scientific (Xi’an, China). Throughout all steps of analysis, deionized water purified by an Ultra Clear System (SG Water Conditioning and Regeneration, Barsbüttel, Germany) was used. Acids and salts of extra-pure grade were from Helicon (Moscow, Russia).

Methyl hydrogen ((((diethoxyphosphoryl)methyl)(ethoxy)phosphoryl)methyl)phosphonate (**3**) was obtained according to [21].

Apo-TKT was purified as previously described in [22] and stored frozen at −20 °C in 50 mM potassium phosphate pH 7.6 with 10% ammonium sulphate. Before use, the buffer was changed to 50 mM glycylglycine pH 7.6 by gel filtration on a Sephadex G-50 column (Pharmacia, Uppsala, Sweden). TKT phosphopentose substrates were synthesized from ribose 5-phosphate as described recently [23].

### 2.2. Preparation of bmThTP

NMR spectra were recorded with the Avance 400 (^1^H 400 MHz, ^13^C 100.6 MHz, ^31^P 161.9 MHz) spectrometer (Bruker, Billerica, MA, USA) at ambient temperature. High-resolution mass spectra were recorded with a G3 QTof quadrupole-time-of-flight from Waters (Milford, MA, USA) equipped with an ESI, which was purchased under the M. V. Lomonosov Moscow State University Program of Development.

The complete scheme of bmThTP synthesis is presented in Section 3.1.

#### 2.2.1. Synthesis of (Z)-4-(N-((4-Amino-2-methylpyrimidin-5-yl)methyl)formamido)-3-(propyldisulfaneyl)pent-3-en-1-yl methyl ((((diethoxyphosphoryl)methyl)(ethoxy)phosphoryl)methyl)phosphonate (**4**)

Diethyl azodicarboxylate (40 wt. % solution in toluene, 1.5 mmol, 3 equiv., 0.69 mL) was added to the solution of triphenylphosphine (1.5 mmol, 3 equiv., 393 mg) in anhydrous tetrahydrofuran (THF) (8 mL) under argon at 0 °C. The reaction mixture was stirred for 1 h at 0 °C and then mixture of methyl hydrogen ((((diethoxyphosphoryl)methyl)(ethoxy)phosphoryl)methyl)phosphonate (**3**, 0.5 mmol, 1 equiv., 183 mg) and N,N-diisopropylethylamine (0.5 mmol, 1 equiv., 87 µL) in 5 ml anhydrous THF and solution of thiamine propyl disulfide (**2**, 0.55 mmol, 1.1 equiv., 196 mg) in 5 ml anhydrous THF were added successively. The resulting reaction mixture was stirred at ambient temperature for 4 h and the solvents were evaporated under reduced pressure. The residue was chromatographed over silica gel (EtOAc/MeOH, 2:1) to yield thiamine propyl disulfide methyl ((((diethoxyphosphoryl)methyl)(ethoxy)phosphoryl)methyl)phosphonate (**4**, 266 mg, 77%) as a colorless viscous oil (mixture of two diastereomers in a ratio of approximately 2:1 (Supplementary Figures S1A–C and S2A)).

Major diastereomer:

^1^H NMR (CDCl_3_, 400 MHz), δ: 7.97 (s, 1H, H-6_(Pyr)_), 7.86 (br.s, 1H, NCHO), 4.28–4.09 (m, 10H, CH_2_N, CH_2_CH_2_OP(O), P(O)OCH_2_CH_3_), 3.81 (d, 11.4 Hz, 3H, P(O)OCH_3_), 3.04–2.95 (m, 2H, CH_2_CH_2_OP(O)), 2.95–2.60 (m, 4H, P(O)CH_2_P(O)), 2.45 (s, 3H, CH_3(Pyr)_), 2.35 (t, *J* 7.1 Hz, 2H, SCH_2_CH_2_CH_3_), 2.07 (s, 3H, CH_3_C=C), 1.54–1.45 (m, 2H, SCH_2_CH_2_CH_3_), 1.40–1.32 (m, 9H, P(O)OCH_2_CH_3_), 0.92 (t, J 7.3 Hz, SCH_2_CH_2_CH_3_).

^13^C NMR (CDCl_3_, 100.6 MHz), δ: 167.6 (C-2_(Pyr)_), 163.5 (NCHO), 162.0 (C-4_(Pyr)_), 156.1 (C-6_(Pyr)_), 133.9 (CH_3_C=C), 133.3 (CH_3_C=C), 108.1 (C-5_(Pyr)_), 63.7 (d, *J* 5.2 Hz, CH_2_CH_2_OP(O)), 62.6–62.0 (m, P(O)(OCH_2_CH_3_)_2_), 61.7–61.4 (m, P(O)OCH_2_CH_3_), 52.9 (d, *J* 5.9 Hz, P(O)OCH_3_), 41.3 (SCH_2_CH_2_CH_3_), 39.9 (CH_2_N), 30.4 (d, *J* 5.9 Hz, CH_2_CH_2_OP(O)), 29.4–26.0 (m, P(O)CH_2_P(O)), 25.3 (CH_3(Pyr)_), 21.8 (SCH_2_CH_2_CH_3_), 18.9 (CH_3_C=C), 16.3–16.0 (m, P(O)OCH_2_CH_3_), 12.7 (SCH_2_CH_2_CH_3_).

^31^P NMR (CDCl_3_, 161.9 MHz), δ: 37.9 (br.s), 22.0 (br.s), 19.6 (br.s).

Minor diastereomer:

^1^H NMR (CDCl_3_, 400 MHz), δ: 7.97 (s, 1H, H-6_(Pyr)_), 7.83 (br.s, 1H, NCHO), 4.28–4.09 (m, 10H, CH_2_N, CH_2_CH_2_OP(O), P(O)OCH_2_CH_3_), 3.85 (d, *J* 11.3 Hz, 3H, P(O)OCH_3_), 3.04–2.95 (m, 2H, CH_2_CH_2_OP(O)), 2.95–2.60 (m, 4H, P(O)CH_2_P(O)), 2.45 (s, 3H, CH_3(Pyr)_), 2.35 (t, *J* 7.1 Hz, 2H, SCH_2_CH_2_CH_3_), 2.06 (s, 3H, CH_3_C=C), 1.54–1.45 (m, 2H, SCH_2_CH_2_CH_3_), 1.40–1.32 (m, 9H, P(O)OCH_2_CH_3_), 0.92 (t, J 7.3 Hz, SCH_2_CH_2_CH_3_).

^13^C NMR (CDCl_3_, 100.6 MHz), δ: 167.7 (C-2_(Pyr)_), 163.4 (NCHO), 162.0 (C-4_(Pyr)_), 156.1 (C-6_(Pyr)_), 133.8 (CH_3_C=C), 133.2 (CH_3_C=C), 108.0 (C-5_(Pyr)_), 63.4 (d, *J* 5.9 Hz, CH_2_CH_2_OP(O)), 62.6–62.0 (m, P(O)(OCH_2_CH_3_)_2_), 61.7–61.4 (m, P(O)OCH_2_CH_3_), 53.3 (d, *J* 6.1 Hz, P(O)OCH_3_), 41.3 (SCH_2_CH_2_CH_3_), 39.9 (CH_2_N), 30.4 (d, *J* 5.9 Hz, CH_2_CH_2_OP(O)), 29.4–26.0 (m, P(O)CH_2_P(O)), 25.3 (CH_3(Pyr)_), 21.8 (SCH_2_CH_2_CH_3_), 18.9 (CH_3_C=C), 16.3–16.0 (m, P(O)OCH_2_CH_3_), 12.7 (SCH_2_CH_2_CH_3_).

^31^P NMR (CDCl_3_, 161.9 MHz), δ: 37.8 (br.s), 21.9 (br.s), 19.5 (br.s).

HRMS (ESI-TOF) calculated for **4**: C_24_H_46_N_4_O_9_P_3_S_2_ [M+H]^+^ 691.1914; found 691.1914.

#### 2.2.2. Synthesis of 3-((4-Amino-2-methylpyrimidin-5-yl)methyl)-5-(2-((hydroxy((hydroxy(phosphonomethyl)phosphoryl)methyl)phosphoryl)oxy)ethyl)-4-methylthiazol-3-ium Chloride Hydrohalide (**1**)

Compound **4** (0.15 mmol, 104 mg) and triphenylphosphine (0.3 mmol, 79 mg) were dissolved in 10 mL dioxane (10 mL), followed by addition of water (2.5 mL) and 1M HCl (1 mL) to adjust pH to 1. The reaction mixture was stirred at ambient temperature for 0.5 h. The solvents were evaporated under vacuum. The residue was dissolved in water (10 mL) and washed three times with dichloromethane (10 mL) and diethyl ether (10 mL). Water was evaporated to dryness to form a mixture of compounds **5a** (two diastereomers) and **5b** (Supplementary Figures S1D–F and S2B), which was used without further purification.

^1^H NMR (CD_3_OD, 400 MHz), δ: 9.88, 9.87, 9.86 (3s, 1H, H-2_(thiazole)_), 8.31, 8.28, 8.26 (3s, 1H, H-6_(Pyr)_), 5.57, 5.56 (m, 2H, CH_2_N^+^), 4.44–4.32, 4.31-4.26 (2m, 2H, CH_2_CH_2_OP(O)), 4.21-4.12 (m, 6H, P(O)OCH_2_CH_3_), 3.82 (d, *J* 11.4 Hz, 1.8 H, P(O)OCH_3_), 3.48–3.37 (m, 2H, CH_2_CH_2_OP(O)), 3.05-2.72 (m, 4H, P(O)CH_2_P(O)), 2.64, 2.63 (2s, 6H, CH_3(thiazole)_, CH_3(Pyr)_), 1.37–1.31 (m, 9H, P(O)OCH_2_CH_3_).

^13^C NMR (D_2_O, 100.6 MHz), δ: 162.9, 162.8 (C-2_(Pyr)_), 162.7, 162.6 (C-4_(Pyr)_), 154.6, 154.4 (C-6_(Pyr)_), 144.6, 144.1 (C-2_(thiazole)_), 143.5, 142.9 (C-4_(thiazole)_), 135.3, 134.2 (C-5_(thiazole)_), 105.7, 105.4 (C-5_(Pyr)_), 65.2-62.6 (m, CH_2_CH_2_OP(O), P(O)OCH_2_CH_3_), 53.7 (d, *J* 6.6 Hz, P(O)OCH_3_), 49.8, 49.6 (CH_2_N^+^), 28.8-24.7 (m, P(O)CH_2_P(O)), 27.5, 27.0 (2 d, *J* 6.6 Hz, *J* 6.8 Hz, CH_2_CH_2_OP(O)), 20.5 (CH_3(Pyr)_), 15.3-15.0 (m, P(O)OCH_2_CH_3_), 10.8, 10.7 (CH_3(thiazole)_).

^31^P NMR (D_2_O, 161.9 MHz), δ: 44.0–43.9 (m), 39.9–39.7 (m), 23.2–23.0 (m), 22.3, 22.2, 21.0, 20.9, 11.6, 11.5.

HRMS (ESI-TOF) calculated for **5a**: C_20_H_38_Cl_2_N_4_O_8_P_3_S [M+H]^+^ 657.0995; not found.

HRMS (ESI-TOF) calculated for **5b**: C_21_H_40_Cl_2_N_4_O_8_P_3_S [M+H]^+^ 671.1151; not found.

The previous mixture of compounds **5a**,**b** was suspended in 15 mL anhydrous dichloromethane and bromotrimethylsilane (1 mmol, 131 µL) was added to a suspension. The reaction mixture was stirred at ambient temperature for 20 h. The solvent was removed under vacuum. The crude residue was dissolved in water (0.5 mL) and reprecipitated from the mixture EtOAc/MeOH (3:1, 8 mL). The sediment was dried under vacuum to yield thiamine bismethylene triphosphate hydrohalide (**1**, 37 mg, 43%) as a white powder with 92% purity based on HPLC and NMR data (Supplementary Figures S1G–I and S2C).

M.p. 160–162 °C.

^1^H NMR (D_2_O, 400 MHz), δ: 9.67 (s, 1H, H-2_(thiazole)_), 7.95 (s, 1H, H-6_(Pyr)_), 5.56 (s, 2H, CH_2_N^+^), 4.18 (dd, *J* 11.6, 5.6 Hz, 2H, CH_2_CH_2_OP(O)), 3.31 (t, *J* 5.6 Hz, 2H, CH_2_CH_2_OP(O)), 2.70–2.46 (m, 4H, P(O)CH_2_P(O)), 2.60 (s, 3H, CH_3(thiazole)_), 2.53 (s, 3H, CH_3(Pyr)_).

^13^C NMR (D_2_O, 100.6 MHz), δ: 162.8 (C-2_(Pyr)_), 162.6 (C-4_(Pyr)_), 154.7 (C-6_(Pyr)_), 143.9 (C-2_(thiazole)_), 143.0 (C-4_(thiazole)_), 135.4 (C-5_(thiazole)_), 106.0 (C-5_(Pyr)_), 63.2 (d, *J* 4.4 Hz, CH_2_CH_2_OP(O)), 49.6 (CH_2_N^+^), 31.6–27.7 (P(O)CH_2_P(O)), 27.4 (d, *J* 6.6 Hz, CH_2_CH_2_OP(O)), 20.6 (CH_3(Pyr)_), 10.8 (CH_3(thiazole)_).

^31^P NMR (D_2_O, 161.9 MHz), δ: 37.1 (br.s), 16.3 (br.s), 15.3 (br.s).

HRMS (ESI-TOF) calculated for **1**: C_14_H_26_Cl_2_N_4_O_8_P_3_S [M+H]^+^ 573.0056; not found, or as C_14_H_26_BrClN_4_O_8_P_3_S [M+H]^+^ 616.9551; not found.

### 2.3. Transketolase Activity Assay

Preincubation of 2–4 µg/mL (for bmThTP) or 1–2 µg/mL (for ThDP) of apo-TKT with the varied concentrations of tested ligands was carried out for 40 min in 50 mM glycylglycine buffer pH 7.6 additionally containing 2.5 mM CaCl_2_. Although most ThDP-dependent enzymes are considered to require Mg^2+^ ions for ThDP binding, Ca^2+^ has been shown to act as a natural cofactor for yeast TKT previously by us [24] and others [25]. Mg^2+^ and other divalent cations may replace Ca^2+^, but this results in lower TKT stability and its affinity towards ThDP. Whereas such long preincubation is required to achieve maximal TKT activity with minimal ThDP concentrations in earlier studies [25], our TKT preparation has been shown not to lose activity even after 24 h in the incubation buffer at room temperature. Negative and positive controls were always included as additional samples preincubated with and without ThDP.

After preincubation, the enzyme (50 µL) was mixed with the TKT assay medium (150 µL), namely 0.25 mM NADH, 4 mg/mL phosphopentose mixture, 13.5 U/mL triosephosphate isomerase, 0.9 U/mL glycerol-3-phosphate dehydrogenase in 50 mM glycylglycine buffer, pH 7.6. NADH oxidation rate was further recorded at 25 °C using CLARIOstarPlus plate reader (BMG LABTECH, Ortenberg, Germany) in spectrophotometric (340 nm) mode for about 30 min [23]. All measurements were reproduced three or four times on different days, and averaged based on these biological replicates; however, all data points were used for the estimation of kinetic parameters determined using Prism, version 8.0 (GraphPad Software Inc., La Jolla, CA, USA).

### 2.4. Molecular Docking

Molecular docking was performed in Gnina v. 1.3 [26] in WSL shell. To simulate the reaction intermediates [27], the coenzyme structures were prepared as hydroxyacetylated derivatives in Chem3D v.18.0 (PerkinElmer Inc., Waltham, MA, USA), and energy was minimized with built-in MM2 and MMFF94 force fields [28]. The negative charges of phosphate groups were assigned manually based on pKa values calculated for ATP and its methylene analogs [29,30]: −3 for ThDP, −4 for ThTP and −3 for bmThTP. The structure of yeast TKT in complex with ThDP, Ca^2+^ and acceptor substrate erythrose-4-phosphate (PDB ID: 1NGS, [31]) was used as a target. The existing ThDP structure was extracted from TKT chain α in a separate .sdf file before docking, together with the removal of water molecules, using PyMOL v.3.0 (Schrödinger, Inc., New York, NY, USA). To ensure the correct positioning of ThDP atoms important in catalysis, as we estimate the coenzyme potential of ThDP analogs and not just their binding, and in view of the identity of two ThDP-binding sites in the TKT homodimer [32], the docking was constrained to one of the active sites. This however does not allow the estimation of cooperativity, known for the two TKT active sites [24].

The ligands were docked into a resulting target structure using the extracted ThDP template and default scoring function implemented in Gnina v. 1.3 together with rescoring using the Monte Carlo method with a Metropolis criterion (--cnn metrorescore). Other parameters were set to default, except for turning off adding hydrogen atoms to ligands (--addH off) and high exhaustiveness (--exhaustiveness 32). Top ligand conformations, i.e., those with the largest CNN scores, were visualized in PyMOL v.3.0 (Schrödinger, Inc., USA). Bond types in between ligand and protein atoms were determined in PLIP Web-server [33] and visualized in LigPlot+ v.2.3.1 [34]. As the default scoring function of Gnina was “linearly reweighted to fit score to free energies (kcal/mol)” [35,36], the output docking scores, i.e., “affinities”, were used as ΔG estimates.

## 3. Results

### 3.1. Synthesis of bmThTP

**Figure 1:**
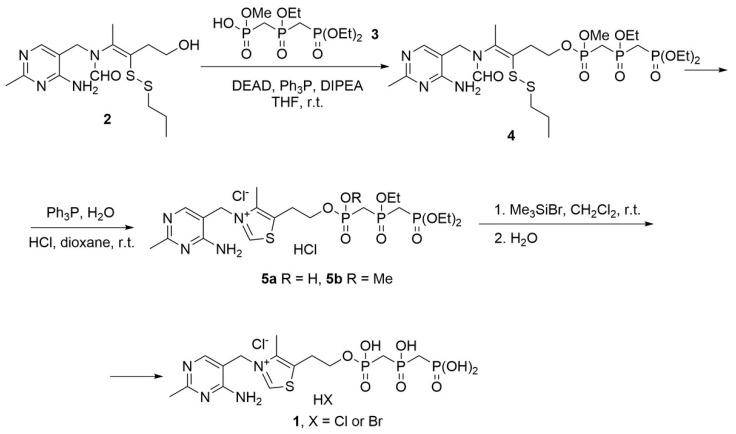
Scheme for bmThTP synthesis. (**1**)—bmThTP, (**2**)—thiamine propyl disulfide, (**3**)—methyl hydrogen ((((diethoxyphosphoryl)methyl)(ethoxy)phosphoryl)methyl)phosphonate, (**4**)—thiamine propyl disulfide methyl ((((diethoxyphosphoryl)methyl)(ethoxy)phosphoryl)methyl)phosphonate, (**5a**,**b**)—esterified bmThTP precursors, DEAD—diethyl azodicarboxylate, Ph_3_P—triphenyl phosphine, DIPEA—diisopropylethylamine, THF—tetrahydrofuran.

The synthesis scheme is presented in Figure 1. The approach to thiamine bismethylene triphosphate (**1**, bmThTP) synthesis was based on an improved procedure described in [37]. Methyl hydrogen ((((diethoxyphosphoryl)methyl)(ethoxy)phosphoryl)methyl)phosphonate (**3**), obtained according to [21], is introduced into the Mitsunobu reaction with thiamine propyl disulfide (**2**). The reaction proceeds smoothly at room temperature; however, it is worth noting that in order to avoid the formation of a salt of thiamine propyl disulfide (**2**) with compound **3**, the addition of diisopropylethylamine is required. Obtained as two diastereomers in 77% yield, disulfide **4** is reduced with triphenyl phosphine followed by cyclization under acidic conditions to a mixture of compounds (**5a,b**) due to the partial hydrolysis of the methoxy group. Final deesterification using bromotrimethylsilane and subsequent reprecipitation results in formation of bmThTP (**1**) in 43% yield.

### 3.2. TKT Can Use bmThTP as the Coenzyme

**Figure 2:**
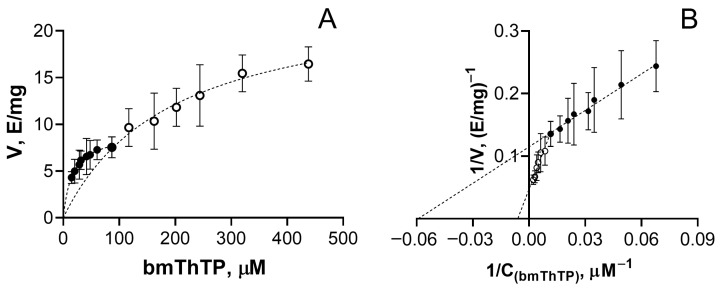
Determination of the apparent K_m_ values for bmThTP towards Ca^2+^-TKT: dependence of TKT activity expressed as E/mg from bmThTP concentration expressed as µM is shown in direct (**A**) and double reciprocal (**B**) coordinates. Kinetic parameters are provided in Table 1. ●—binding in first high-affinity sites, ○—binding in second low-affinity sites. The dashed lines represent Michaelis-Menten (**A**) or linear (**B**) approximations of data points.

The ThTP analog bmThTP does possess the coenzyme function, as revealed by the appearance of TKT activity when bmThTP was added to purified apo-TKT (Figure 2). Importantly, no TKT activity was observed when the esterified precursors of bmThTP (**5a**,**b**) were added to apo-TKT up to a 900 µM concentration. Thus, the TKT activity with bmThTP (Figure 2) cannot be attributed to contamination from its synthesis. On the other hand, bmThTP is a hydrolysis-resistant analog of ThTP, and the synthesis itself does not use ThDP (Figure 1). So, any chance that ThDP could be responsible for the TKT activity is excluded. Thus, bmThTP can act as a TKT coenzyme in vitro.

As expected, comparison of TKT kinetic parameters for bmThTP and ThDP suggests a higher ThDP affinity (Table 1). That is, K_m_ ^1^ (bmThTP), i.e., apparent K_m_ for high-affinity sites, is 362-fold higher than the corresponding K_m_ ^1^ (ThDP), and 42-fold higher than K_m_ ^2^ (ThDP). Both V_max_ (bmThTP) values are approximately two times lower than the corresponding V_max_ (ThDP). Notably, the 10-fold difference between K_m_ ^2^ (ThDP) and K_m_ ^1^ (ThDP) is also preserved for bmThTP, suggesting the same cooperative mechanism for the appearance of the second type of the active sites within the TKT dimer upon binding of the first coenzyme molecule [24]. Thus, the ability of bmThTP to act as the TKT coenzyme in vitro should be considered as a proof of concept, with the stable synthetic ligand being used as a model of natural hydrolysis-susceptible ThTP.

### 3.3. Comparison of ThDP, ThTP, and bmThTP Binding to the TKT Active Site

The hydrolysis-resistant bmThTP is indispensable for the study of ThTP coenzyme function, which cannot be analyzed directly due to the interference from ThDP upon ThTP hydrolysis. Assuming similar binding parameters for ThTP and bmThTP, hydrolysis of even 1% of ThTP would significantly affect TKT reaction in vitro (Table 1). However, substitution of two methylene groups in bmThTP (Figure 1) instead of ThTP oxygen atoms can influence the ligand binding significantly, for example by disrupting H-bond(s) formed by the ThTP phosphates within the TKT active site. Fortunately, a hydrolysis-independent comparison of all the three ligands can be made in silico using molecular docking.

The modeling results show that bmThTP binds substantially worse than ThTP, with the three ligands’ calculated affinities being in the order of ThDP > ThTP >> bmThTP (Table 2). Moreover, the differences in estimated ΔG values suggest the ThTP binding constant is much closer to that of ThDP than bmThTP. Using experimentally determined TKT K_m_ ^1^ (Table 1) and predicted ΔG values (Table 2), the K_m_ ^1^ (ThTP) can be estimated to be 0.07–0.08 µM (Table 2). The calculations are well supported by the experimentally determined K_m_ ^1^ values for both bmThTP and ThDP. Thus, although K_m_ ^1^(ThTP) cannot be measured directly, it is predicted to be approximately 0.07–0.08 µM, which is only 1.6–2 times higher than K_m_ ^1^(ThDP) (Table 1 and Table 2).

**Figure 3:**
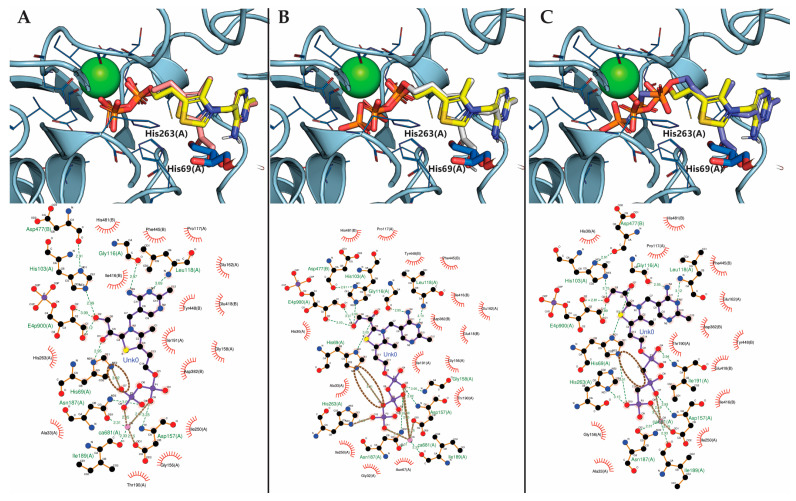
Predicted poses (**top panels**) of hydroxyacetylated TKT coenzymes bound in the active site of the enzyme and visualization of their interactions with TKT and other TKT ligands (**bottom panels**). (**A**) ThDP, (**B**) ThTP and (**C**) bmThTP were introduced in TKT (PDB ID: 1NGS, [31]) using molecular docking. Carbon atoms of TKT chains α and β are slate-colored. Carbon atoms of ThDP initially present in 1NGS model are yellow-colored and those of substrate (erythrose-4-phosphate) are marine-colored. The residues interacting with the phosphate/phosphonate moieties of ThDP/ThTP/bmThTP are shown as wires. Carbons atoms of docked coenzymes are shown in pink (ThDP), gray (ThTP) or deep slate (bmThTP). Calcium ion is shown as a green sphere. Other atoms are shown using the standard color code. Dashed green lines on LigPlot-generated [34] bottom panels indicate H-bonds, thicker brown lines indicate salt bridges (ionic bonds), according to PLIP Web-server [33]. Ionic bond between ThDP (**A**) or ThTP (**B**) and His69 of TKT, absent in bmThTP (**C**), is circled.

The calculations are also supported by the comparison of the predicted conformations of the TKT ligands bound to the enzyme active site (Figure 3A–C). Ligand interactions and bonds of the ThDP position determined by X-ray crystallography [31], and of ThDP, ThTP, and bmThTP positions modeled by molecular docking, can be easily visualized using PyMOL (Figure 3, top panels) or LigPlot+ (Figure 3, bottom panels). Comparison of these models show that negatively charged phosphate groups of ThDP and ThTP form double salt bridges, i.e., ionic bonds, with Ca^2+^ ions as well as His263 and His69 residues of the TKT chain α (Figure 3A,B). No such bonds can be observed when phosphate groups are replaced with the phosphonate ones in bmThTP. According to PLIP Web-server [33], the binding of the latter results in a slightly different orientation of the β-phosphonate group of the coenzyme and weaker H-bonds with both His residues (Figure 3C). The lack of an ionic bond between His69 residue and oxygen atom linking β- and γ-phosphates (Figure 3A,B) absent in the ThTP analog (Figure 3C) seems to be the key factor determining the weaker binding of bmThTP. Thus, although the position of the reactive C2 atom of the thiazole ring is virtually the same for the three ligands, bonding of bmThTP to the TKT active site is less tight, compared to ThTP, which is reflected in higher predicted binding energies (Table 2).

## 4. Discussion

As mentioned above, in the case of ThTP accumulation, its fraction of the total thiamine content can substantially exceed that of ThDP. For example, such cases were reported for a highly specialized muscle-like tissue in the electric organs of several fishes. ThTP concentration of 3.9 nmol/g wet weight accounted for 87% of total thiamine, exceeding the ThDP level 8.5-fold in *Electrophorus electricus* [11]. Also, ThTP concentration of 45 nmol/g wet weight (38% of total thiamine) was five times higher compared to ThDP concentration (only 8% of total thiamine) in *Torpedo marmorata* [38]. Among birds, extreme ThTP accumulation was found in white skeletal muscles of chicken, where ThTP concentration of 7.06 nmol/g wet weight corresponded to 81% of total thiamine content [13]. In mammals, a large amount of ThTP was found in pig skeletal muscles. In this tissue, ThTP concentration of 26.1 nmol/g wet weight equaled 69% of total thiamine on average, with up to 89% in some samples [12]. Thus, ThTP concentration may exceed that of ThDP up to 9-fold in muscles of different animals, including mammals. Moreover, while ThTP concentrations of approximately 4–45 µM has been reported in muscle and related tissues, its intracellular concentrations may exceed those values due to compartmentalization.

Upon accumulation, ThTP was detected almost solely in cytosol [11,12]. It was also associated with muscle cytosolic adenylate kinase activity, however the determined K_m_(ThDP) for the main muscle adenylate kinase isoenzyme, encoded by the *AK1* gene, exceeded 2 mM, suggesting another enzyme to be most likely involved in ThTP production [13]. This was further supported by *AK1* knockout in mice, which resulted in muscle ThTP content being similar to the wild-type mice [39]. With that kept in mind, the mechanism itself remains a potent source for ThTP production, especially taking into account seven more adenylate kinase isoenzymes encoded in mammalian genomes [3]. In addition to the cytosolic synthesis, ThTP can be produced from ThDP in rat brain mitochondria [40] or *Arabidopsis thaliana* chloroplasts [41] by ATP synthase. Such ThTP can later be exported from the mitochondrial matrix in exchange for phosphate or nucleotides in energized mitochondria [40]. Although two distinct mechanisms of ThTP synthesis have been shown, the accumulation of ThTP is usually associated with a lack of THTPA activity. The presence of the latter enzyme seems to be restricted to animals [10], with birds having lost the enzyme completely [42], while fish and domestic pig enzymes are catalytically inefficient [10,19,42,43]. Thus, elevated ThTP may act primarily on cytosolic targets and TKT appears to be the only ThDP-dependent enzyme with primary cytosolic localization [18].

Using the hydrolysis-resistant ThTP analog bmThTP, here we show its ability to act as a TKT coenzyme in vitro, and estimate the K_m_ ^1^(ThTP) value as 71–80 nM—only two times higher than that of ThDP (Table 2). Although more computation-heavy approaches, like molecular dynamics simulations, are expected to improve the predictions of ΔG and K_m_ values, such approaches are not flawless and are not guaranteed to surpass the docking scoring functions [44]. This is especially true for TKT, as its active site is located at the interface between two subunits and involves metal ions (Ca^2+^) in the binding of the coenzymes. Moreover, our docking scores (Table 2) are in good accordance with the around 300-400-fold difference in K_i_ values determined for triazole–ThDP and its methylene analog towards another ThDP-dependent enzyme, pyruvate decarboxylase from *Z. mobilis* [45], although these compounds act as irreversible inhibitors, not coenzymes.

Since ThTP concentration can exceed that of ThDP up to 9-fold, given its twofold higher K_m_ values, it could thus even substitute ThDP in TKT active sites. Taking into account the accumulated data on ThTP concentrations in different species [43], the calculated K_m_ ^1^ (ThTP) appears to be within the physiological range, not just in birds, fishes and pigs. For example, submicromolar-to-micromolar concentrations of ThTP have been reported in brewer’s yeast (*Saccharomyces carlsbergensis*), slugs (*Arion rufus*) or baboons (*Papio papio*). ThTP can also temporally accumulate up to micromolar concentrations as a stress response in *E. coli* [10]. One can propose that TKT activity would be reduced upon ThTP binding to the active sites of the enzyme based on the two-times-lower V_max_ of bmThTP compared to ThDP (Table 1). The latter may be physiologically relevant for the pentose phosphate pathway flux, since TKT is the rate-limiting enzyme of its non-oxidative branch [46,47]. The pathway is essential for antioxidant defense and the lipid metabolism of muscle cells, especially during muscle repair and regeneration [48,49]. However, such a direct assumption is speculative, and requires special experiments studying the impact of ThTP accumulation in animal tissues on their physiology and metabolism. Thus, we propose that the ability of ThTP to act as a physiologically relevant coenzyme of the ThDP-dependent enzyme TKT implies there is no special need to rescue the coenzyme by THTPA as previously suggested [50]. This also explains the absence of physiological complications of ThTP accumulation in animals devoid of active THTPA such as pigs or chickens.

## 5. Conclusions

Using a novel hydrolysis-resistant ThTP analog, bmThTP, we show its ability to act as a coenzyme of TKT in vitro. Obtained kinetic parameters of bmThTP and ThDP and affinities of bmThTP, ThTP and ThDP to TKT calculated with molecular docking enable the estimation of K_m_ ^1^(ThTP), whose direct evaluation is interfered with by spontaneous hydrolysis of ThTP. The Km value estimated as 71–80 nM suggests that ThTP could act as a physiologically relevant TKT coenzyme in addition to ThDP in animals upon conditions of extreme ThTP accumulation. Such cases of ThTP concentrations substantially exceeding that of ThDP are reported for several species devoid of active ThTP-hydrolyzing enzyme cytosolic THTPA. Thus, on one hand our data propose the ability of ThTP to act as a coenzyme similar to ThDP. The latter extends our knowledge about this ubiquitous thiamine triphosphorylated derivative which lacks a clear physiological role. However, on the other hand, it stimulates the discussion on the physiological role of THTPA and/or ThTP in animals and other species requiring novel experiments and a special attention to the physiological consequences of accumulated ThTP, which likely possesses both the coenzyme and signaling potential.

## Figures and Tables

**Table 1:** Kinetic parameters for TKT with the studied coenzymes. Indices ^1^ and ^2^ correspond to the two binding sites with high (1) and low (2) affinities to the coenzymes.

	K_m_ ^1^ (µM)	K_m_ ^2^ (µM)	V_max_ ^1^ (E/mg)	V_max_ ^2^ (E/mg)
bmThTP	16.3 ± 8.6	194.3 ± 97.2	9.1 ± 1.5	24.0 ± 5.2
ThDP *	0.045 ± 0.005	0.39 ± 0.03	19.8 ± 1.1	59.3 ± 2.2
ThDP **	0.036 ± 0.003	0.38 ± 0.04	15 ± 3	69 ± 17

*—Data obtained here; **—data from [24].

**Table 2:** Estimation of kinetic parameters for TKT with ThDP, ThTP, and bmThTP as coenzymes. The differences in ΔG values predicted from molecular docking and experimentally determined K_m_ ^1^ (ThDP) and K_m_ ^1^ (bmThTP) (Table 1) are used for the estimation of the calculated K_m_^1^ values for other ligands. The parameters calculated based on the K_m_ ^1^ (bmThTP) are shown in brackets in order to be distinguished from the ones calculated using K_m_ ^1^ (ThDP). n.a.—not applicable.

	TKT Parameters		Differences with ThDP (bmThTP)					
	K_m_ ^1^ (µM)	Predicted ΔG (kcal/mol)	Predicted ΔG (kcal/mol)		K_m_ ^1^ Fold Change		Calculated K_m_ ^1^ (µM)	
ThDP	0.045	−10.54	−	(−2.61)	−	(407)	−	(0.040)
ThTP	n.a.	−10.29	+0.25	(−2.36)	1.78	(229)	0.080	(0.071)
bmThTP	16.3	−7.93	+2.61	−	407	−	18.3	−

## Data Availability

Dataset available on request from the authors.

## Supplementary Materials

The following supporting information can be downloaded at: https://www.mdpi.com/article/10.3390/biom16020304/s1, Figure S1: NMR spectra observed for bmThTP (compound **1**) and its precursors (compounds **4** and **5**a-b); Figure S2: HRMS (ESI-TOF) spectra observed for bmThTP (compound **1**) and its precursors (compounds **4** and **5**a-b).

## Abbreviations

The following abbreviations are used in this manuscript:

ThDP	thiamine diphosphate, or thiamine pyrophosphate, or cocarboxylase
ThTP	thiamine triphosphate
bmThTP	bismethylene ThTP
TKT	Transketolase
THTPA	cytosolic thiamine triphosphatase
PDH	pyruvate dehydrogenase
OGDH	2-oxoglutarate dehydrogenase, or α-ketoglutarate dehydrogenase
BCDH	branched-chain 2-oxo acid dehydrogenase
HACL	2-hydroxyacyl-CoA-lyase
THF	tetrahydrofuran

## References

[1] Makarchikov A.F., Wins P., Bettendorff L. (2025). Biochemical and medical aspects of vitamin B1 research. Neurochem. Int..

[2] Zhang K., Bian J., Deng Y., Smith A., Nunez R.E., Li M.B., Pal U., Yu A.-M., Qiu W., Ealick S.E. (2016). Lyme disease spirochaete Borrelia burgdorferi does not require thiamin. Nat. Microbiol..

[3] Aleshin V.A., Mkrtchyan G.V., Bunik V.I. (2019). Mechanisms of Non-coenzyme Action of Thiamine: Protein Targets and Medical Significance. Biochem. Biokhimiia.

[4] Bergmeyer H.U., Bergmeyer H.U. (1974). Methods of Enzymatic Analysis.

[5] Kiessling K.-H. (1961). Thiamine triphosphate in liver and brain of the rat. Biochim. Biophys. Acta.

[6] Kiessling K.-H. (1956). Studies of thiamine triphosphate. Biochim. Biophys. Acta.

[7] De La Fuente G., DÍAz-Cadavieco R. (1954). Cocarboxylasic Activity of Thiamine Phosphoric Esters. Nature.

[8] Rossi-Fanelli A., Siliprandi N., Siliprandi D., Ciccarone P. (1955). Triphosphothiamine. I. Preparation and crystallization of the pure compound. Some chemical and enzymatic properties. Arch. Biochem. Biophys..

[9] Bettendorff L., Lakaye B., Kohn G., Wins P. (2014). Thiamine triphosphate: A ubiquitous molecule in search of a physiological role. Metab. Brain Dis..

[10] Bettendorff L. (2021). Update on Thiamine Triphosphorylated Derivatives and Metabolizing Enzymatic Complexes. Biomolecules.

[11] Bettendorff L., Michel-Cahay C., Grandfils C., Rycker C.D., Schoffeniels E. (2006). Thiamine Triphosphate and Membrane-Associated Thiamine Phosphatases in the Electric Organ of Electrophorus electricus. J. Neurochem..

[12] Egi Y., Koyama S., Shikata H., Yamada K., Kawasaki T. (1986). Content of thiamin phosphate esters in mammalian tissues--an extremely high concentration of thiamin triphosphate in pig skeletal muscle. Biochem. Int..

[13] Miyoshi K., Egi Y., Shioda T., Kawasaki T. (1990). Evidence for In Vivo Synthesis of Thiamin Triphosphate by Cytosolic Adenylate Kinase in Chicken Skeletal Muscle. J. Biochem..

[14] Plokhikh K.S., Nesterov S.V., Chesnokov Y.M., Rogov A.G., Kamyshinsky R.A., Vasiliev A.L., Yaguzhinsky L.S., Vasilov R.G. (2023). Association of 2-oxoacid dehydrogenase complexes with respirasomes in mitochondria. FEBS J..

[15] Quinlan C.L., Goncalves R.L., Hey-Mogensen M., Yadava N., Bunik V.I., Brand M.D. (2014). The 2-oxoacid dehydrogenase complexes in mitochondria can produce superoxide/hydrogen peroxide at much higher rates than complex I. J. Biol. Chem..

[16] Fraccascia P., Casteels M., De Schryver E., Van Veldhoven P.P. (2011). Role of thiamine pyrophosphate in oligomerisation, functioning and import of peroxisomal 2-hydroxyacyl-CoA lyase. Biochim. Biophys. Acta.

[17] Kitamura T., Seki N., Kihara A. (2017). Phytosphingosine degradation pathway includes fatty acid alpha-oxidation reactions in the endoplasmic reticulum. Proc. Natl. Acad. Sci. USA.

[18] Li M., Zhang X., Lu Y., Meng S., Quan H., Hou P., Tong P., Chai D., Gao X., Zheng J. (2020). The nuclear translocation of transketolase inhibits the farnesoid receptor expression by promoting the binding of HDAC3 to FXR promoter in hepatocellular carcinoma cell lines. Cell Death Dis..

[19] Szyniarowski P., Lakaye B., Czerniecki J., Makarchikov A.F., Wins P., Margineanu I., Coumans B., Grisar T., Bettendorff L. (2005). Pig tissues express a catalytically inefficient 25-kDa thiamine triphosphatase: Insight in the catalytic mechanisms of this enzyme. Biochim. Biophys. Acta (BBA) Gen. Subj..

[20] Lacabanne D., Wiegand T., Wili N., Kozlova M.I., Cadalbert R., Klose D., Mulkidjanian A.Y., Meier B.H., Bockmann A. (2020). ATP Analogues for Structural Investigations: Case Studies of a DnaB Helicase and an ABC Transporter. Molecules.

[21] Taylor S.D., Mirzaei F., Bearne S.L. (2006). An Unsymmetrical Approach to the Synthesis of Bismethylene Triphosphate Analogues. Org. Lett..

[22] Solovjeva O.N. (2002). Isolation and Properties of Noncovalent Complex of Transketolase with RNA. Biochemistry.

[23] Aleshin V., Borisova N., Artiukhov A., Tagirov K., Solovjeva O., Lavrenteva E., Panin N., Maslova M., Graf A. (2025). Daytime-Dependent Effects of Thiamine on the Thiamine Pool and Pyruvate Dehydrogenase Regulation in the Brain and Heart. Int. J. Mol. Sci..

[24] Solovjeva O.N., Selivanov V.A., Orlov V.N., Kochetov G.A. (2019). Stages of the formation of nonequivalence of active centers of transketolase from baker’s yeast. Mol. Catal..

[25] Heinrich P.C., Steffen H., Janser P., Wiss O. (1972). Studies on the reconstitution of apotransketolase with thiamine pyrophosphate and analogs of the coenzyme. Eur. J. Biochem..

[26] McNutt A.T., Li Y., Meli R., Aggarwal R., Koes D.R. (2025). GNINA 1.3: The next increment in molecular docking with deep learning. J. Cheminform..

[27] Fiedler E., Thorell S., Sandalova T., Golbik R., König S., Schneider G. (2002). Snapshot of a key intermediate in enzymatic thiamin catalysis: Crystal structure of the α-carbanion of (α,β-dihydroxyethyl)-thiamin diphosphate in the active site of transketolase from Saccharomyces cerevisiae. Proc. Natl. Acad. Sci. USA.

[28] Artiukhov A.V., Aleshin V.A. (2025). Covalent Docking to the Active Sites of Thiamine Diphosphate-Dependent Enzymes. Molecules.

[29] Myers T.C., Nakamura K., Flesher J.W. (2002). Phosphonic Acid Analogs of Nucleoside Phosphates. I. The Synthesis of 5″-Adenylyl Methylenediphosphonate, a Phosphonic Acid Analog of ATP. J. Am. Chem. Soc..

[30] Chan W.M., Welch W., Sitsapesan R. (2003). Structural Characteristics That Govern Binding to, and Modulation through, the Cardiac Ryanodine Receptor Nucleotide Binding Site. Mol. Pharmacol..

[31] Nilsson U., Meshalkina L., Lindqvist Y., Schneider G. (1997). Examination of Substrate Binding in Thiamin Diphosphate- dependent Transketolase by Protein Crystallography and Site-directed Mutagenesis. J. Biol. Chem..

[32] Mitschke L., Parthier C., Schroder-Tittmann K., Coy J., Ludtke S., Tittmann K. (2010). The crystal structure of human transketolase and new insights into its mode of action. J. Biol. Chem..

[33] Schake P., Bolz S.N., Linnemann K., Schroeder M. (2025). PLIP 2025: Introducing protein–protein interactions to the protein–ligand interaction profiler. Nucleic Acids Res..

[34] Laskowski R.A., Swindells M.B. (2011). LigPlot+: Multiple Ligand–Protein Interaction Diagrams for Drug Discovery. J. Chem. Inf. Model..

[35] Koes D.R. Molecular Docking with GNINA 1.0. https://gnina.github.io/gnina/rsc_workshop2021/.

[36] McNutt A.T., Francoeur P., Aggarwal R., Masuda T., Meli R., Ragoza M., Sunseri J., Koes D.R. (2021). GNINA 1.0: Molecular docking with deep learning. J. Cheminform..

[37] Klein E., Nghiêm H.-O., Valleix A., Mioskowski C., Lebeau L. (2002). Synthesis of Stable Analogues of Thiamine Di- and Triphosphate as Tools for Probing a New Phosphorylation Pathway. Chem. Eur. J..

[38] Eder L., Dunant Y. (2006). Thiamine and Cholinergic Transmission in the Electric Organ of Torpedo. J. Neurochem..

[39] Makarchikov A.F., Wins P., Janssen E., Wieringa B., Grisar T., Bettendorff L. (2002). Adenylate kinase 1 knockout mice have normal thiamine triphosphate levels. Biochim. Biophys. Acta (BBA) Mol. Cell Res..

[40] Gangolf M., Wins P., Thiry M., El Moualij B., Bettendorff L. (2010). Thiamine Triphosphate Synthesis in Rat Brain Occurs in Mitochondria and Is Coupled to the Respiratory Chain. J. Biol. Chem..

[41] Wang K., Alric J., Fitzpatrick T.B. (2025). Thiamine triphosphate puts the brake on the activation state of chloroplast ATP synthase. Plant Physiol..

[42] Delvaux D., Kerff F., Murty M.R.V.S., Lakaye B., Czerniecki J., Kohn G., Wins P., Herman R., Gabelica V., Heuze F. (2013). Structural determinants of specificity and catalytic mechanism in mammalian 25-kDa thiamine triphosphatase. Biochim. Biophys. Acta (BBA) Gen. Subj..

[43] Makarchikov A.F., Lakaye B., Gulyai I.E., Czerniecki J., Coumans B., Wins P., Grisar T., Bettendorff L. (2003). Thiamine triphosphate and thiamine triphosphatase activities: From bacteria to mammals. Cell. Mol. Life Sci. (CMLS).

[44] Hou T., Wang J., Li Y., Wang W. (2011). Assessing the performance of the molecular mechanics/Poisson Boltzmann surface area and molecular mechanics/generalized Born surface area methods. II. The accuracy of ranking poses generated from docking. J. Comput. Chem..

[45] Erixon K.M., Dabalos C.L., Leeper F.J. (2007). Inhibition of pyruvate decarboxylase from Z. mobilis by novel analogues of thiamine pyrophosphate: Investigating pyrophosphate mimics. Chem. Commun..

[46] Solovjeva O.N. (2021). The mechanism of a one-substrate transketolase reaction. Part II. Anal. Biochem..

[47] Jia D., Liu C., Zhu Z., Cao Y., Wen W., Hong Z., Liu Y., Liu E., Chen L., Chen C. (2022). Novel transketolase inhibitor oroxylin A suppresses the non-oxidative pentose phosphate pathway and hepatocellular carcinoma tumour growth in mice and patient-derived organoids. Clin. Transl. Med..

[48] Hoshino D., Kawata K., Kunida K., Hatano A., Yugi K., Wada T., Fujii M., Sano T., Ito Y., Furuichi Y. (2020). Trans-omic Analysis Reveals ROS-Dependent Pentose Phosphate Pathway Activation after High-Frequency Electrical Stimulation in C2C12 Myotubes. iScience.

[49] García-Domínguez E., Carretero A., Viña-Almunia A., Domenech-Fernandez J., Olaso-Gonzalez G., Viña J., Gomez-Cabrera M.C. (2022). Glucose 6-P Dehydrogenase—An Antioxidant Enzyme with Regulatory Functions in Skeletal Muscle during Exercise. Cells.

[50] Linster C.L., Van Schaftingen E., Hanson A.D. (2013). Metabolite damage and its repair or pre-emption. Nat. Chem. Biol..

