# Supplementary material for: Investigating Coenzyme Function of Thiamine Triphosphate Using Its Novel Hydrolysis-Resistant Analog and Transketolase
Source: Biomolecules. 2026 Feb 14;16(2):304. doi: 10.3390/biom16020304 (PMC12937740; doi:10.3390/biom16020304)
Supplement: Supplementary file 1 [file biomolecules-16-00304-s001.zip › biomolecules-4099025-supplementary.pdf]

biomolecules

MDPI

Supplementary Materials

Investigating Coenzyme Function of Thiamine Triphosphate Using Its Novel Hydrolysis-Resistant Analog and Transketolase

Artem V. Artiukhov [1,2,†], Alexey V. Kazantsev [3,†], Olga N. Solovjeva [1,*] and Vasily A. Aleshin [1,2,*]

[1] Belozersky Institute of Physico-Chemical Biology, Lomonosov Moscow State University, 19991 Moscow, Russia

[2] Department of Biological Chemistry, Sechenov University, 119048 Moscow, Russia;

[3] Faculty of Chemistry, Lomonosov Moscow State University, 119234 Moscow, Russia; mak@org.chem.msu.ru

† These authors contributed equally to this work and share first authorship;

* Correspondence: aleshinvasily@gmail.com; soloveva_o@list.ru; Tel.: +7 (495) 939-44-84.

Supplementary Figures

A

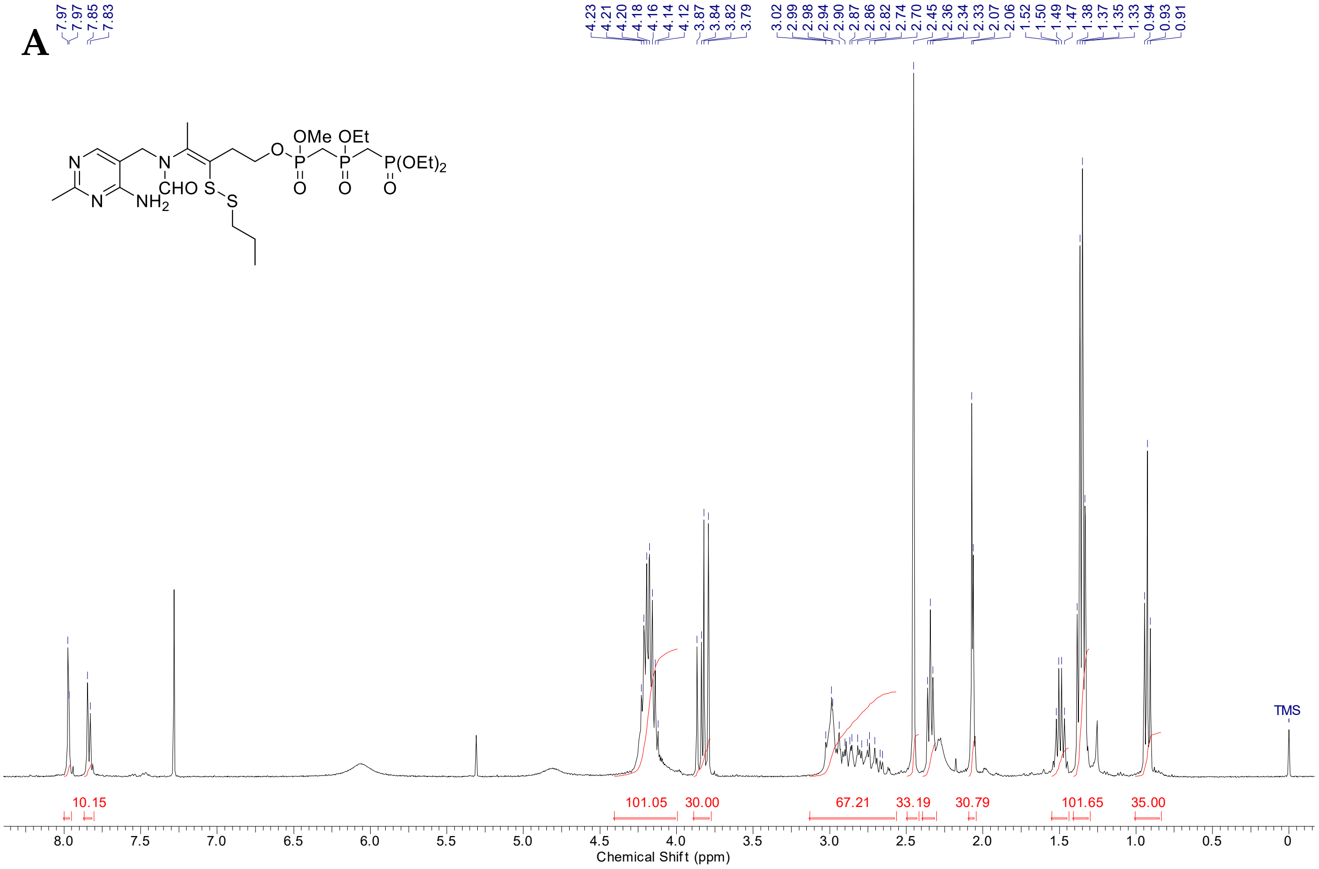

B

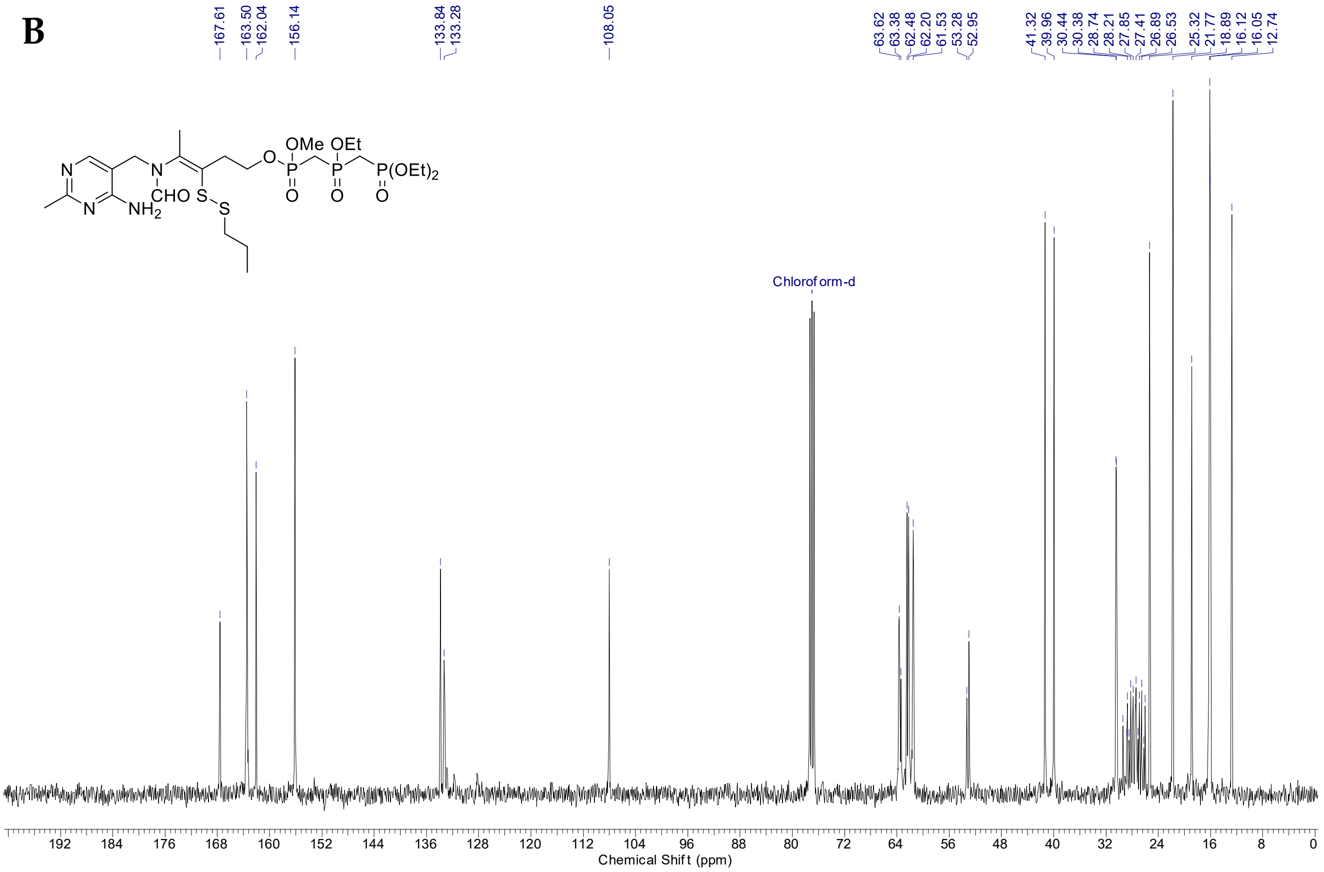
167.61
163.50
162.04
156.14
133.84
133.28
108.05
63.62
63.38
62.48
62.20
61.53
53.28
52.95
41.32
39.96
30.44
30.38
28.74
28.21
27.85
27.41
26.89
26.53
25.32
21.77
18.89
16.12
16.05
12.74
OMe OEt
P(OEt)2
CHO
NH2
Chloroform-d
Chemical Shift (ppm)

C

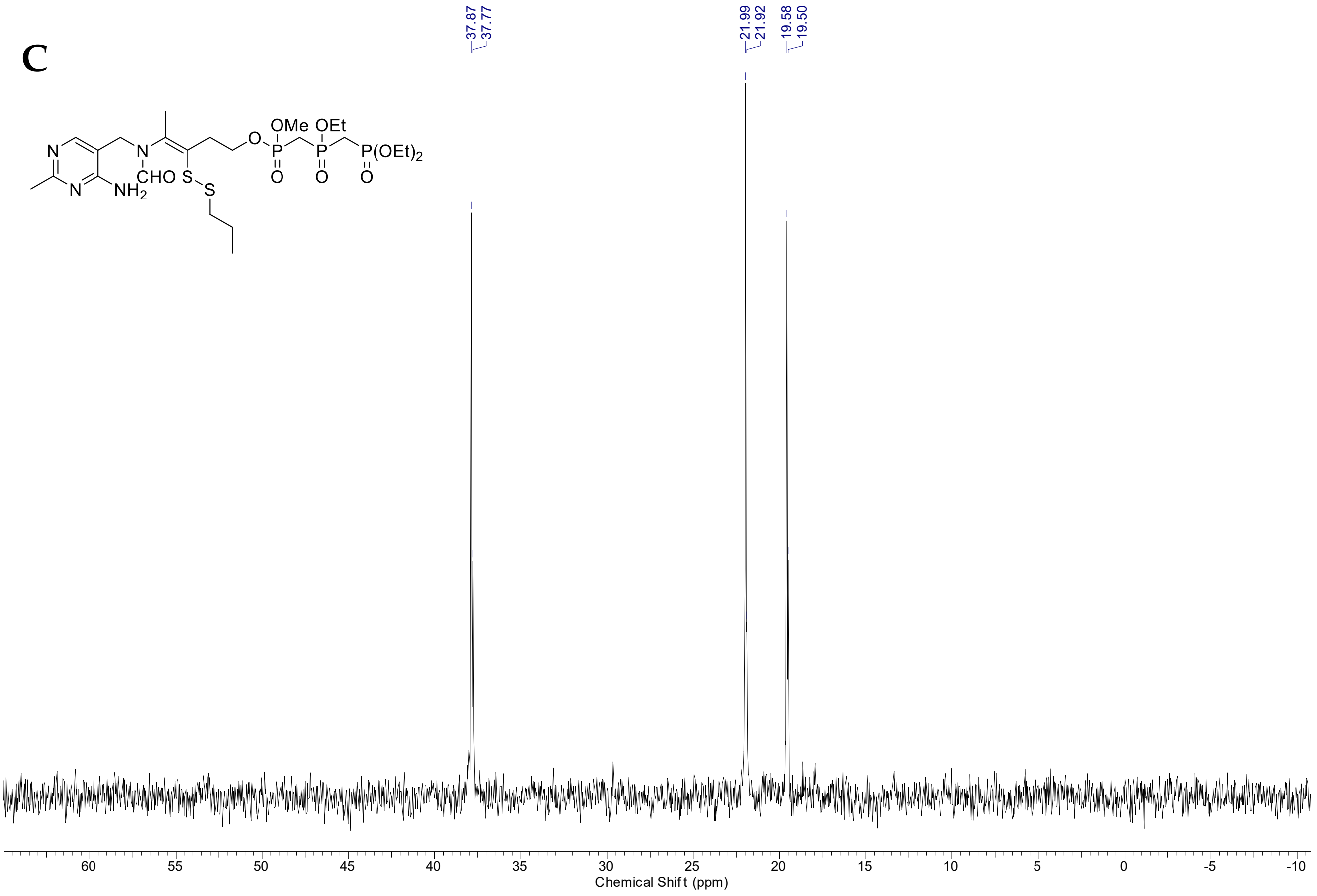
37.87
37.77
21.99
21.92
19.58
19.50
OMe OEt
P(OEt)2
CHO
NH2
Chemical Shift (ppm)

D

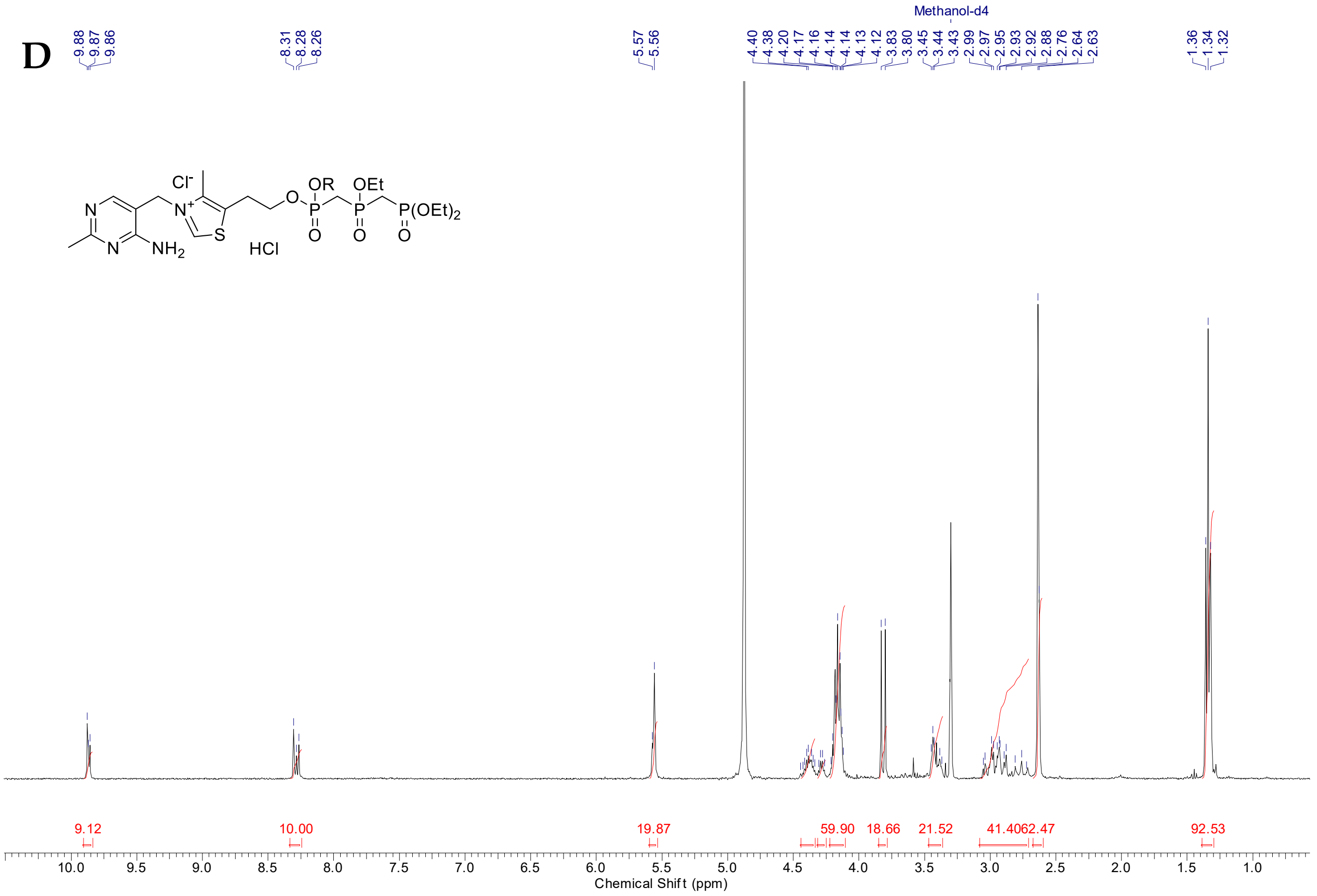

E

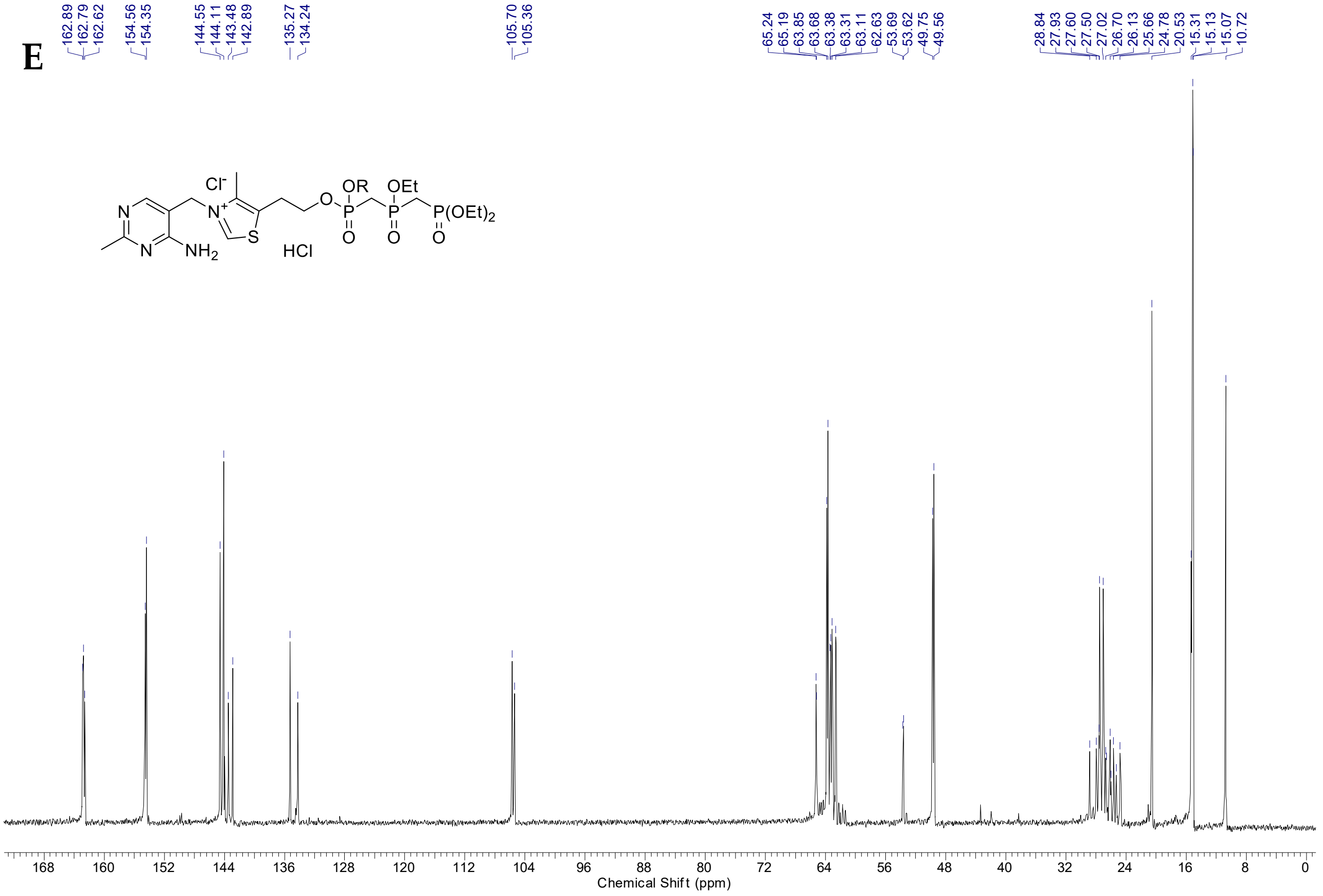

F

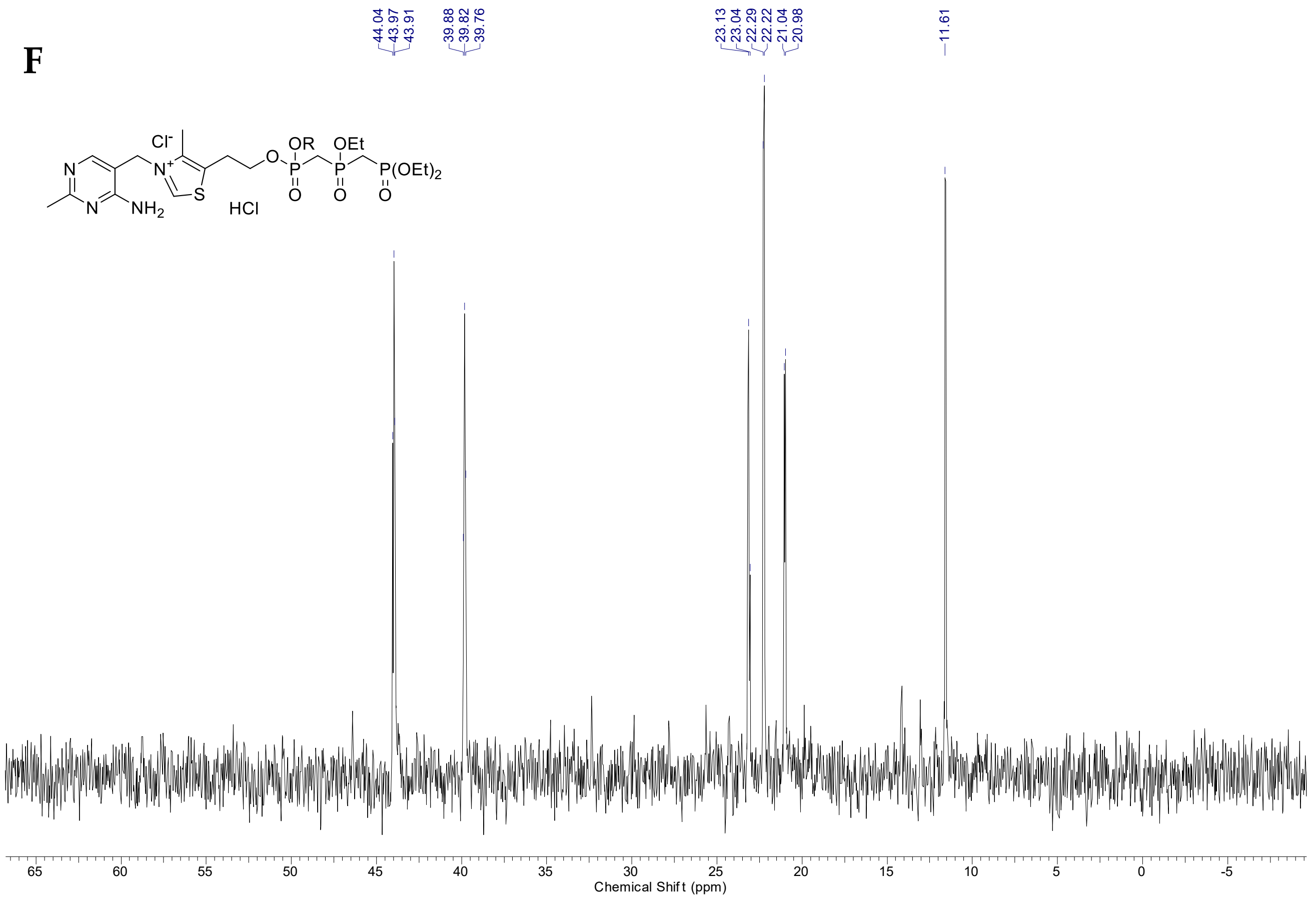
44.04
43.97
43.91
39.88
39.82
39.76
23.13
23.04
22.29
22.22
21.04
20.98
11.61
Cl-
OR
OEt
N
N+
O
P
P
P(OEt)2
S
O
O
O
N
NH2
HCl
65 60 55 50 45 40 35 30 25 20 15 10 5 0 -5
Chemical Shift (ppm)

G

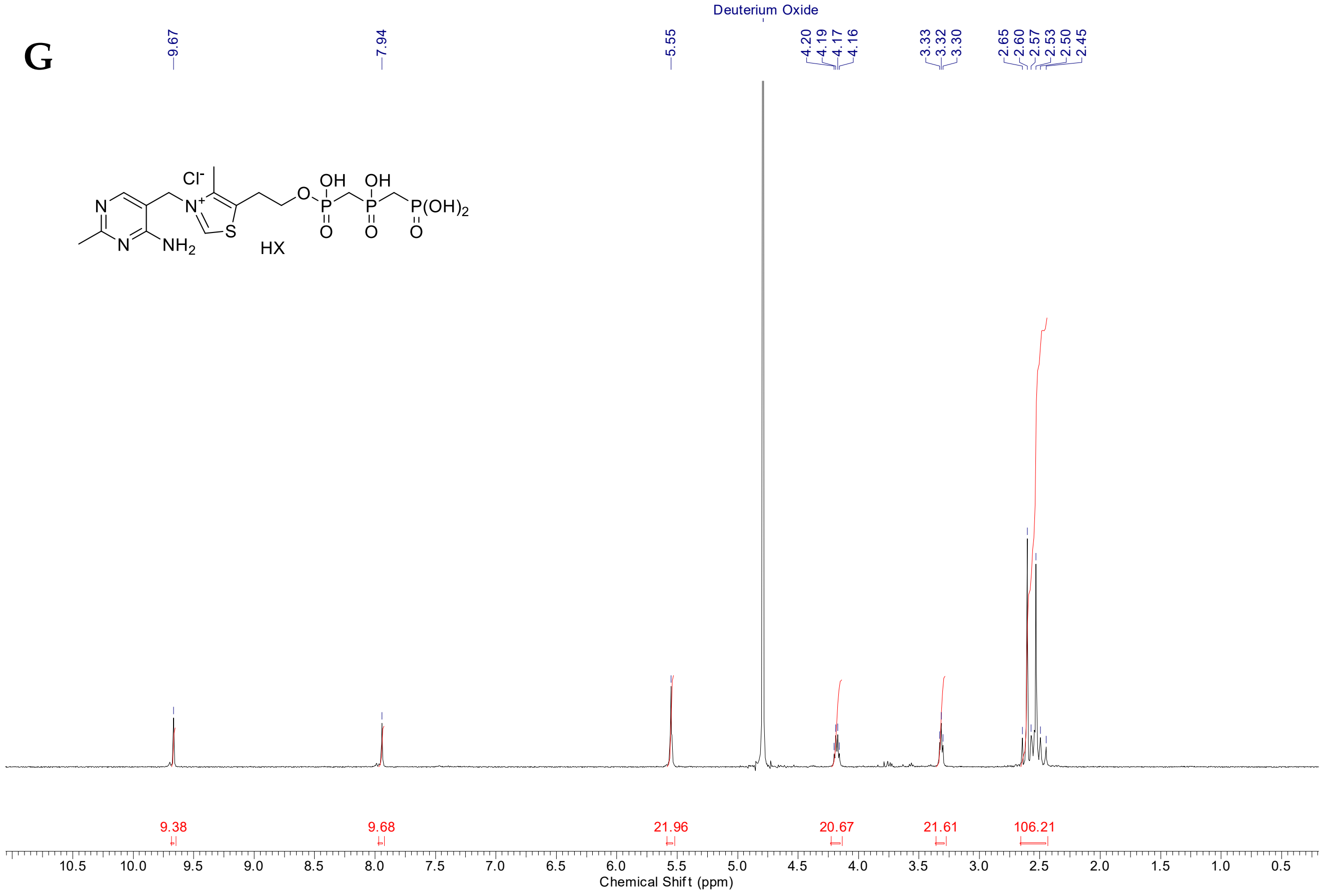
Deuterium Oxide
9.67
7.94
5.55
4.20
4.19
4.17
4.16
3.33
3.32
3.30
2.65
2.60
2.57
2.53
2.50
2.45
Cl-
OH
OH
N
N+
O
P
P
P(OH)2
S
O
O
O
N
NH2
HX
9.38
9.68
21.96
20.67
21.61
106.21
10.5 10.0 9.5 9.0 8.5 8.0 7.5 7.0 6.5 6.0 5.5 5.0 4.5 4.0 3.5 3.0 2.5 2.0 1.5 1.0 0.5
Chemical Shift (ppm)

H

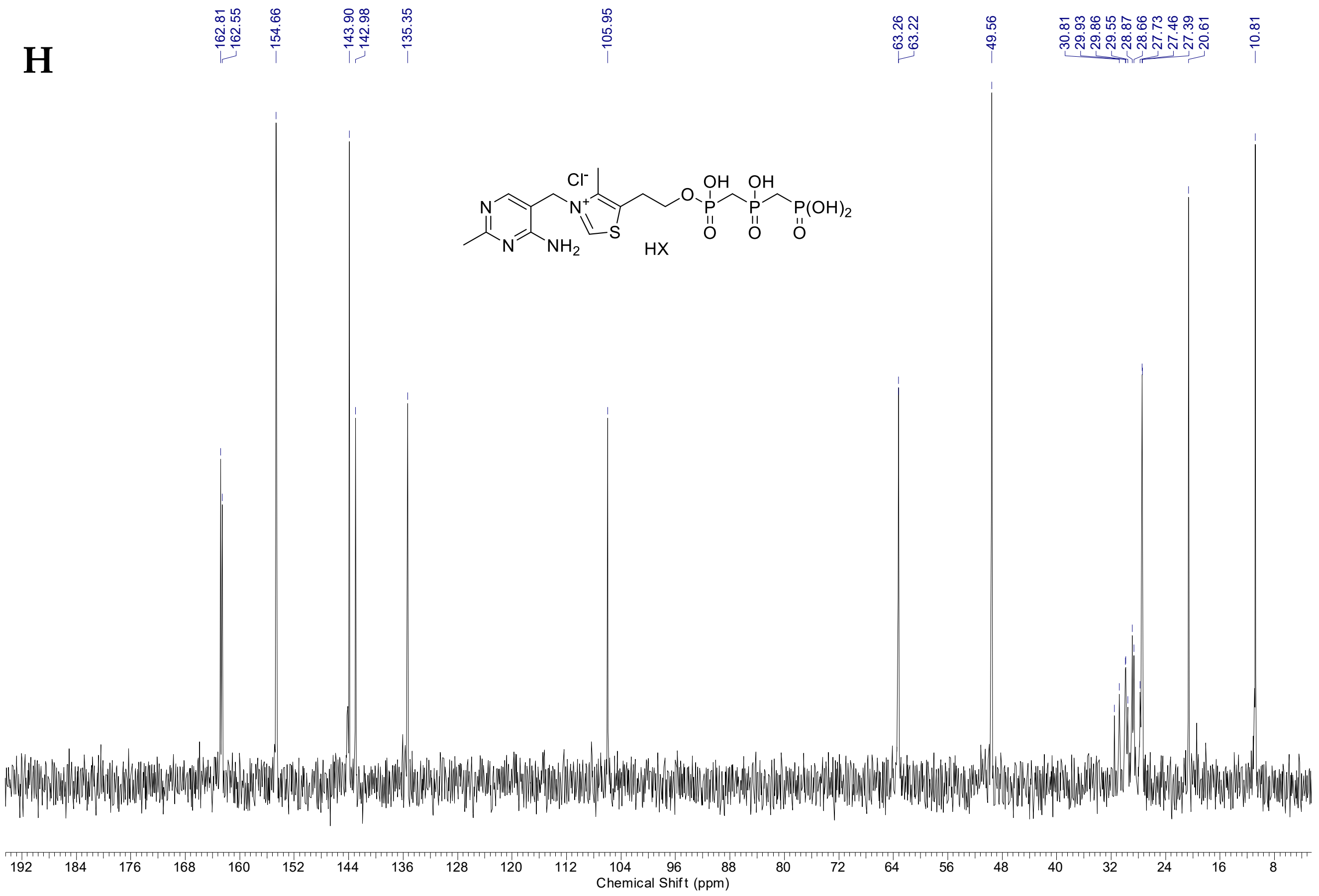
162.81
162.55
154.66
143.90
142.98
135.35
105.95
63.26
63.22
49.56
30.81
29.93
29.86
29.55
28.87
28.66
27.73
27.46
27.39
20.61
10.81
Cl-
N+
S
N
N
NH2
OH
OH
P
P
P(OH)2
O
O
O
O
HX
Chemical Shift (ppm)

I

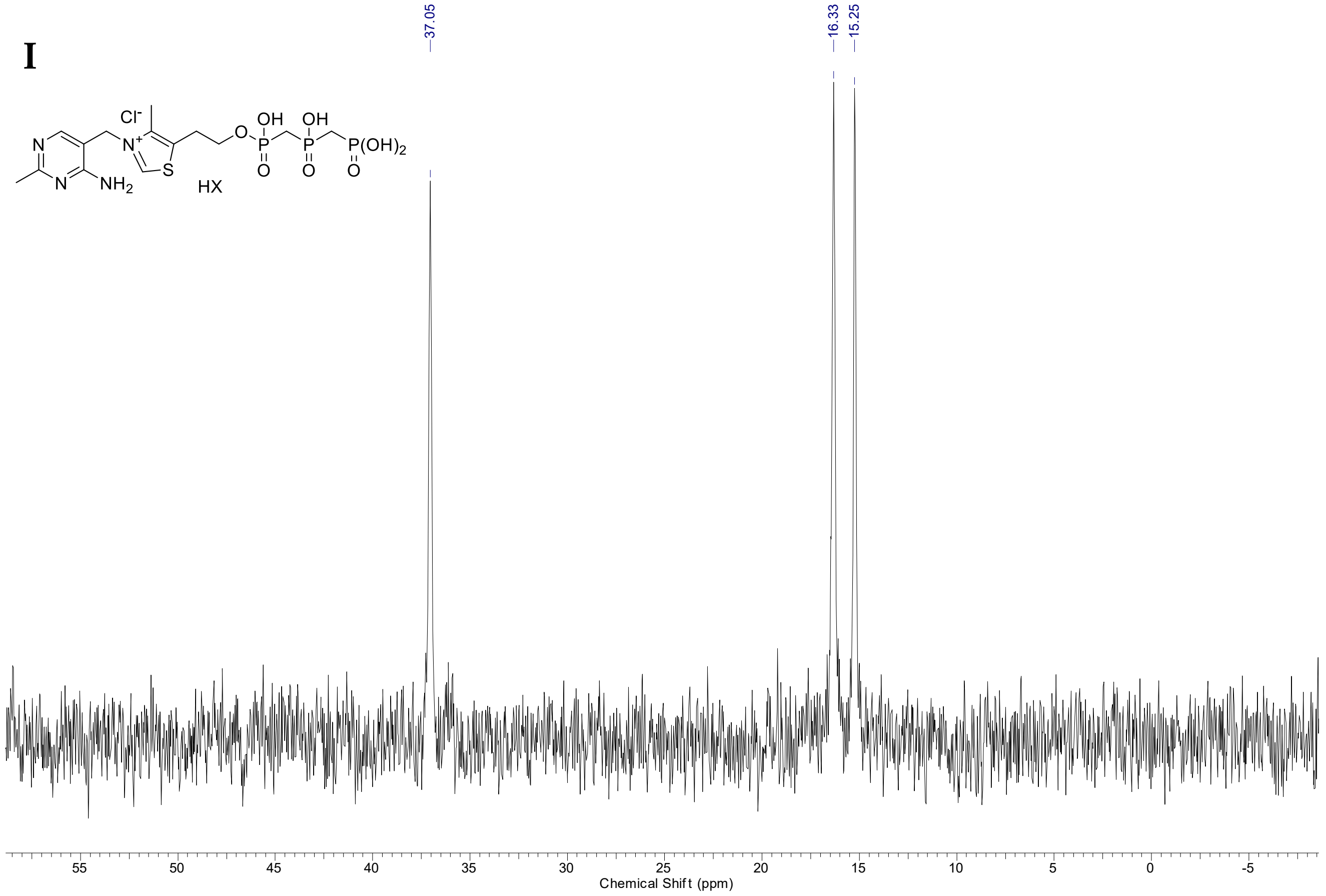
37.05
16.33
15.25
Cl-
N+
S
N
N
NH2
OH
OH
P
P
P(OH)2
O
O
O
O
HX
Chemical Shift (ppm)

Supplementary Figure S1. NMR spectra observed for bmThTP (compound 1) and its precursors (compounds 4 and 5a-b). (**A**) ^{1}H spectra for compound 4. (**B**) ^{13}C spectra for compound 4. (**C**) ^{31}P spectra for compound 4. (**D**) ^{1}H spectra for compounds 5a-b. (**E**) ^{13}C spectra for compounds 5a-b. (**F**) ^{31}P spectra for compounds 5a-b. (**G**) ^{1}H spectra for compound 1. (**H**) ^{13}C spectra for compound 1. (**I**) ^{31}P spectra for compound 1.

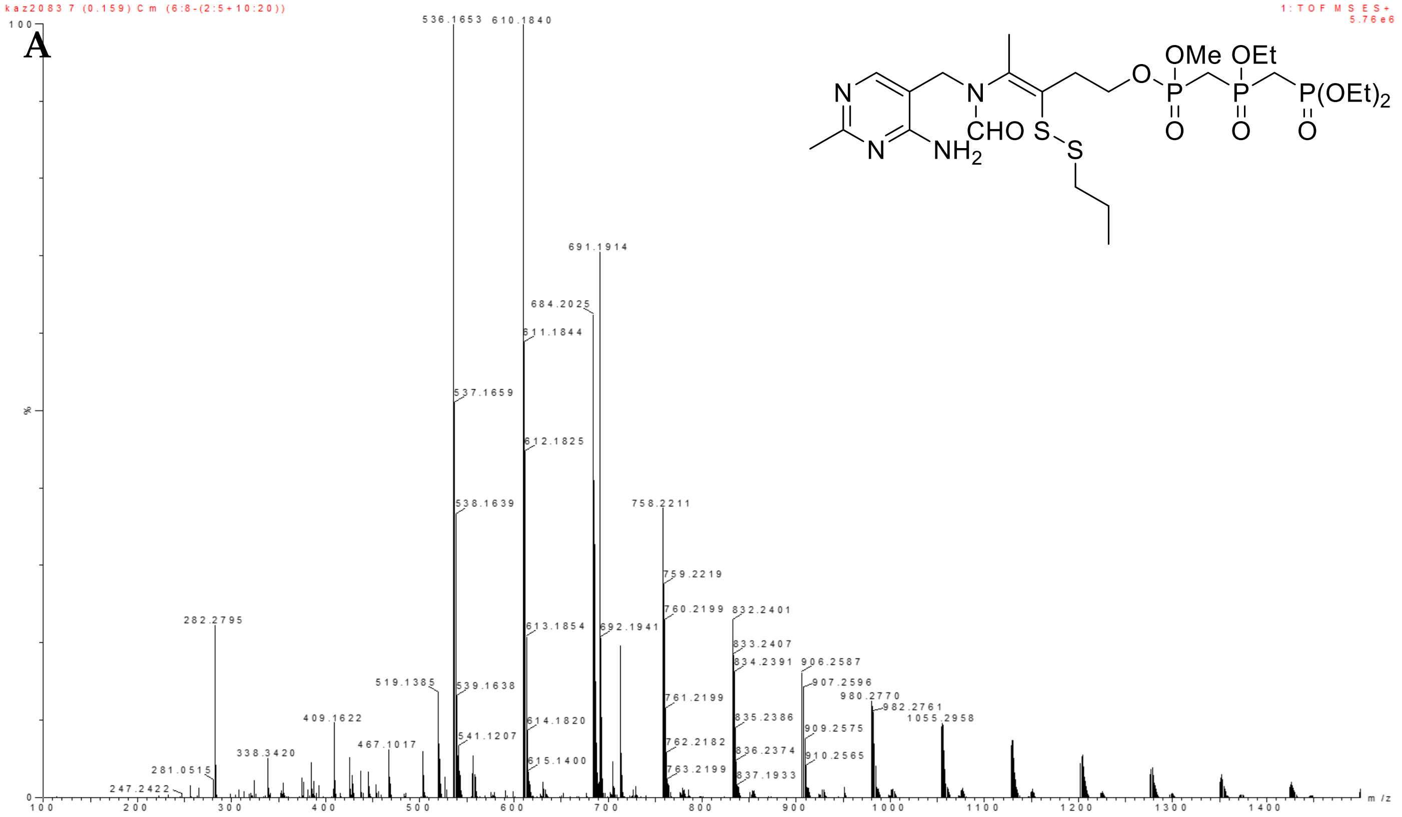
kaz2083 7 (0.159) Cm (6:8-(2:5+10:20))
1: TOF MS ES+
5.76e6
A
OMe
OEt
N
N
NH2
CHO
S
S
P
P
P(OEt)2
O
536.1653
610.1840
691.1914
684.2025
611.1844
537.1659
612.1825
538.1639
758.2211
759.2219
760.2199
832.2401
282.2795
613.1854
692.1941
833.2407
834.2391
906.2587
519.1385
539.1638
907.2596
980.2770
982.2761
1055.2958
409.1622
761.2199
614.1820
835.2386
541.1207
909.2575
338.3420
467.1017
762.2182
836.2374
910.2565
615.1400
281.0515
763.2199
837.1933
247.2422
m/z
%

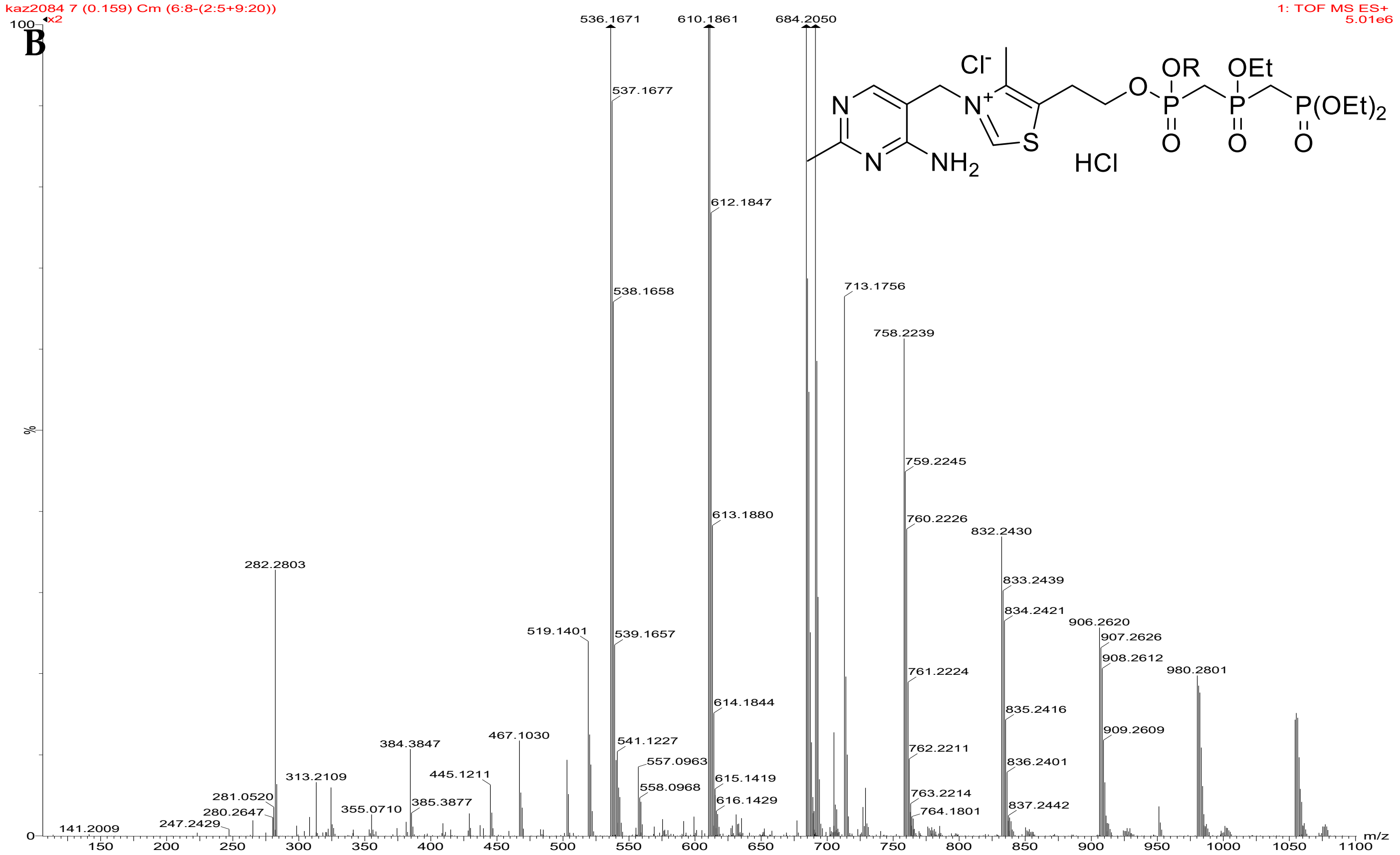
kaz2084 7 (0.159) Cm (6:8-(2:5+9:20))
1: TOF MS ES+
5.01e6
x2
B
Cl-
OR
OEt
N
N+
N
NH2
S
HCl
P
P
P(OEt)2
O
536.1671
610.1861
684.2050
537.1677
612.1847
538.1658
713.1756
758.2239
759.2245
613.1880
760.2226
832.2430
282.2803
833.2439
834.2421
906.2620
907.2626
908.2612
519.1401
539.1657
980.2801
761.2224
614.1844
835.2416
909.2609
467.1030
541.1227
384.3847
762.2211
445.1211
557.0963
836.2401
615.1419
313.2109
558.0968
281.0520
616.1429
763.2214
280.2647
355.0710
385.3877
837.2442
764.1801
247.2429
141.2009
m/z
%

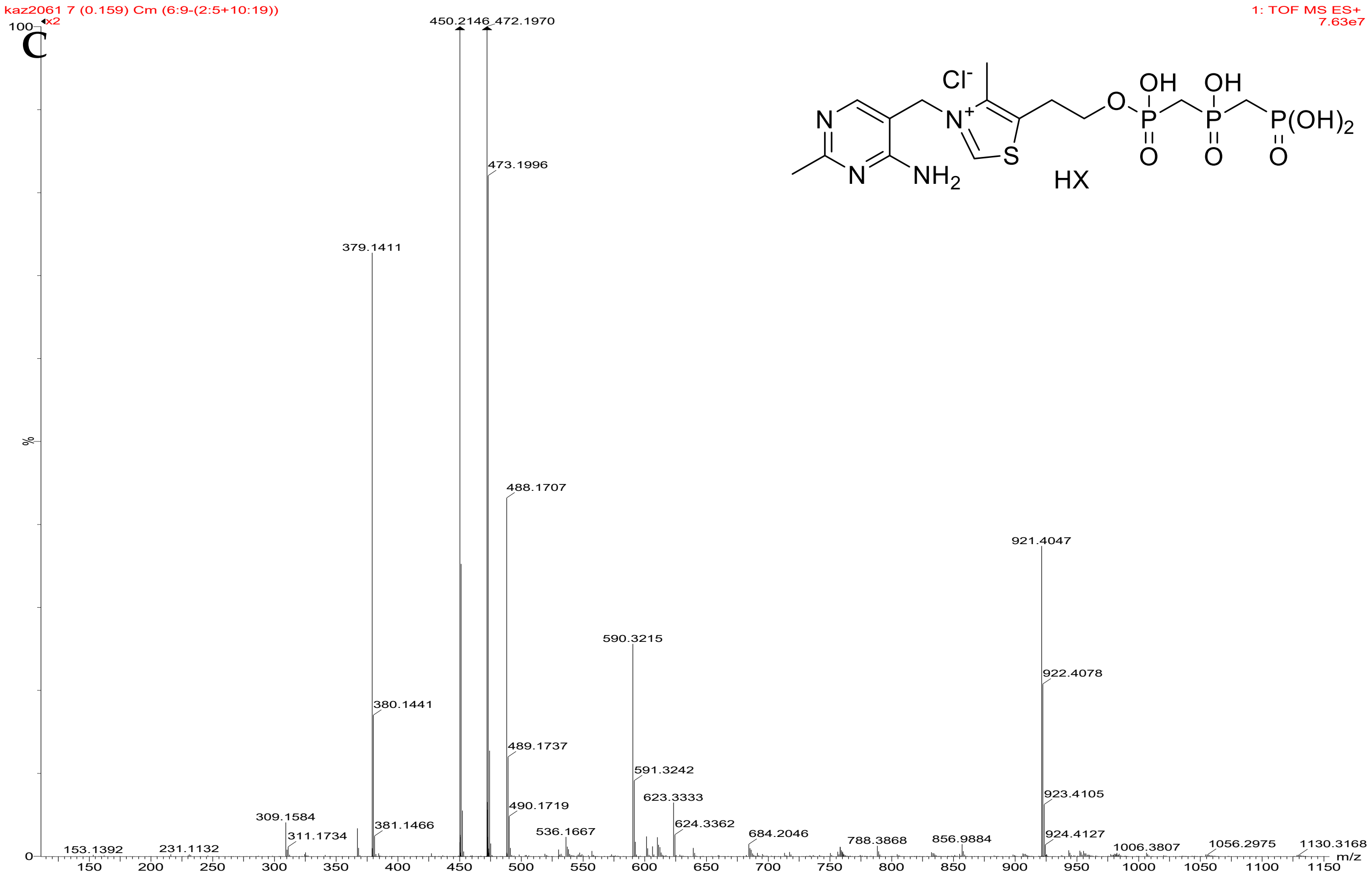

Supplementary Figure S2. HRMS (ESI-TOF) spectra observed for bmThTP (compound 1) and its precursors (compounds 4 and 5a-b). (**A**) Compound 4. (**B**) Compounds 5a-b. (**C**) Compound 1.

Disclaimer/Publisher's Note: The statements, opinions and data contained in all publications are solely those of the individual author(s) and contributor(s) and not of MDPI and/or the editor(s). MDPI and/or the editor(s) disclaim responsibility for any injury to people or property resulting from any ideas, methods, instructions or products referred to in the content.
